# Gas-Phase Nitrous Acid (HONO) Is Controlled by Surface Interactions of Adsorbed Nitrite (NO_2_^–^) on Common Indoor Material Surfaces

**DOI:** 10.1021/acs.est.2c02042

**Published:** 2022-08-24

**Authors:** Shubhrangshu Pandit, Vicki H. Grassian

**Affiliations:** Department of Chemistry and Biochemistry, University of California San Diego, La Jolla, California 92093, United States

**Keywords:** indoor surfaces, nitrous acid (HONO) formation, nitrogen dioxide (NO_2_) hydrolysis, surface reactions, nitrate (NO_3_^−^) photochemistry, surface nitrite

## Abstract

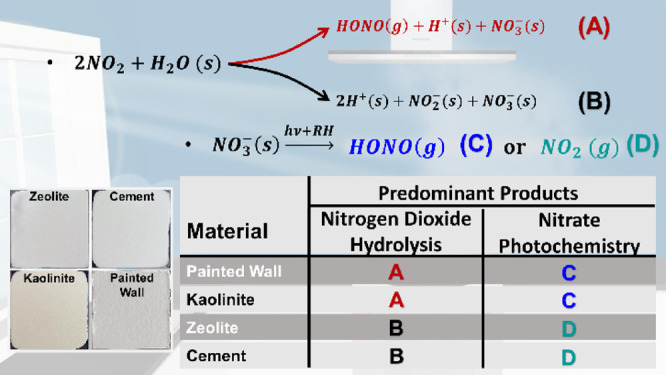

Nitrous acid (HONO) is a household pollutant exhibiting adverse health effects and a major source of indoor OH radicals under a variety of lighting conditions. The present study focuses on gas-phase HONO and condensed-phase nitrite and nitrate formation on indoor surface thin films following heterogeneous hydrolysis of NO_2_, in the presence and absence of light, and nitrate (NO_3_^–^) photochemistry. These thin films are composed of common building materials including zeolite, kaolinite, painted walls, and cement. Gas-phase HONO is measured using an incoherent broadband cavity-enhanced ultraviolet absorption spectrometer (IBBCEAS), whereby condensed-phase products, adsorbed nitrite and nitrate, are quantified using ion chromatography. All of the surface materials used in this study can store nitrogen oxides as nitrate, but only thin films of zeolite and cement can act as condensed-phase nitrite reservoirs. For both the photo-enhanced heterogeneous hydrolysis of NO_2_ and nitrate photochemistry, the amount of HONO produced depends on the material surface. For zeolite and cement, little HONO is produced, whereas HONO is the major product from kaolinite and painted wall surfaces. An important result of this study is that surface interactions of adsorbed nitrite are key to HONO formation, and the stronger the interaction of nitrite with the surface, the less gas-phase HONO produced.

## Abstract 2

HONO concentrations in indoor environments, produced from the heterogeneous hydrolysis of nitrogen dioxide and nitrate surface photochemistry, are controlled by the material surface interactions especially as it relates to the stability of adsorbed nitrite.

## Introduction

The influence of indoor air quality on human health is gaining increasing interest given it is estimated that people spent 80 to 90% of their time indoors.^1^ Modern building constructions are motivated by energy efficiency, lower running costs, and minimal environmental impacts.^2^ Lower air exchange rates and recirculation of air result in enhancement of the level of pollutants generated indoors and can greatly exceed the outdoor concentration.^2−4^

Nitrous acid (HONO) is an important household pollutant with an average indoor concentration of 5–10 ppb.^5,6^ HONO indoor mixing ratios can be elevated up to 90 ppb through combustion while using gas stoves, space heaters, and open fireplaces.^6−8^ HONO can give rise to health risks due to its toxicity, acidity, aqueous solubility, and high reactivity.^9^ HONO can produce carcinogenic molecules such as nitrosamines, known as third-hand smoke, through reaction with surface deposited nicotine and organic amines.^10^ In an indoor environment, photochemistry of HONO by direct sunlight as well as indoor light sources is predicted to contribute up to two orders of magnitude higher indoor OH radical concentration compared to alkene ozonolysis and NO + HO_2_ reactions.^11^ Therefore, there is great interest in understanding indoor HONO chemistry and the factors controlling it.

The correlation between HONO and NO_2_ in different indoor studies indicates that a process involving NO_2_ is the source of HONO.^11−13^ As a result, heterogeneous hydrolysis of NO_2_ on surface is considered as an important source of indoor HONO.^1,2^ In addition, there have been studies that have shown enhanced NO_2_ uptake on surfaces and concomitant HONO production in the presence of light λ < 400 nm.^14−18^ Several indoor relevant solid materials and solutions such as TiO_2_ containing white paint, gypsum, solid organic compounds, lacquer, and acidic bathroom cleaner have been examined for photo-enhanced NO_2_ uptake followed by gas-phase HONO production.^15−19^ Photochemical HONO production involves either photolysis of nitrate or electron transfer to NO_2_ from a photoexcited system such as TiO_2_ or unsaturated organics. Carslaw et al. predicted that the indoor surface to volume ratio is up to 300 times higher than those for outdoors in their model study where these surfaces can act as both the sink and source of gas-phase pollutants.^4^ Collins et al. reported that direct conversion of NO_2_ to HONO has a weak influence on the indoor HONO mixing ratio, suggesting that surface species (adsorbed NO_2_^–^ and HONO) form and gas-phase HONO is controlled strongly by gas-surface equilibrium.^20^ To better understand this multiphase chemistry, a comparative study of HONO production from NO_2_ hydrolysis and nitrate photochemistry has been carried out on four different indoor relevant surface materials: white paint, a mixture of CaO + CaCO_3_ as a cement proxy, zeolite, and kaolinite. TiO_2_-containing photocatalytic paints are used to eliminate the indoor gas-phase pollutants such as NO_*x*_, SO_*x*_, NH_3_, CO, and volatile organic compounds.^21,22^ Previous studies predicted that painted surfaces effectively reduce NO_2_ to HONO, which is enhanced with the increasing wall temperature and in the presence of sunlight or indoor relevant lights.^12,19^ In this study, painted wall surfaces are examined as a potential source and sink of indoor HONO. Cement is used as a binder in concrete, a mixture of calcium oxide (CaO) and calcium carbonate (CaCO_3_), representing a large part of indoor surfaces.^2^ In this study, we used a mixture of CaO and CaCO_3_ as a proxy for cement. The most popular cement is made through the calcination of limestone (CaCO_3_).^23^ This process is a major contributor to global CO_2_ emissions.^23^ In recent years, much effort has been put to reduce the required amount of cement in concrete to make lightweight concrete for both economic and environmental reasons. Natural zeolite and kaolinite are suitable raw materials as a partial substitute for Portland cement. These aluminosilicate materials can adsorb and remove several pollutants. Engineered zeolites are good selective catalytic reduction materials for NO_*x*_ removal in diesel emissions.^24^ These three different materials are compared to a painted surface as a potential source and sink of indoor HONO.

In particular, we investigated the heterogeneous hydrolysis of NO_2_, in the presence and absence of light, and nitrate photochemistry on different indoor surface materials. Gas-phase (HONO and NO_2_) and condensed-phase (NO_3_^–^ and NO_2_^–^) products are quantified using cavity-enhanced ultraviolet (UV)-absorption spectroscopy and ion chromatography, respectively. This approach of simultaneous measurements of gas and condensed phases provided important insights into the multiphase equilibrium of HONO(g)/NO_2_^–^(s) in an indoor air environment. This comparative study shows clearly that surface interactions of adsorbed nitrite determine the extent to which HONO is released to the gas phase.

## Materials and Methods

### Materials

**Figure 1:**
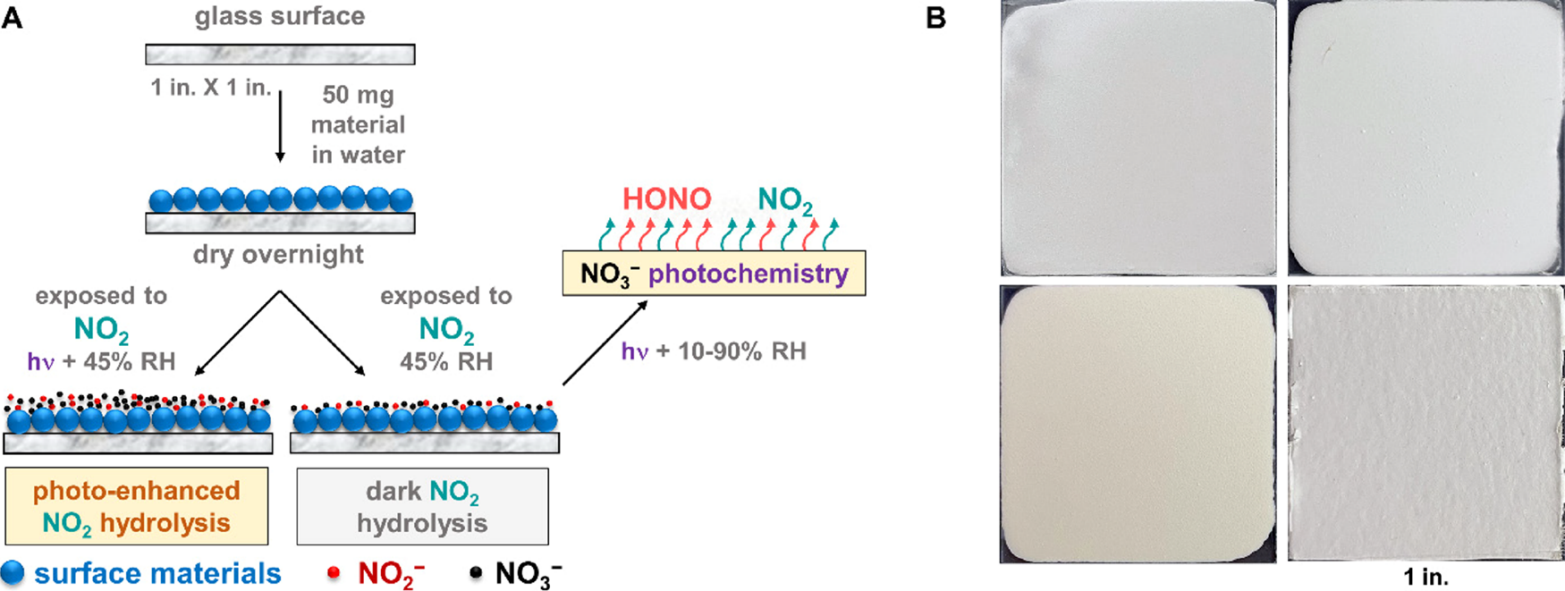
(A) Schematic diagram to illustrate thin film preparation and the different experiments done in this study. NO_2_ exposure under humid conditions in the dark (dark NO_2_ hydrolysis) and under solar illumination (photo-enhanced NO_2_ hydrolysis) and photochemistry of surface adsorbed nitrate (NO_3_^–^ photochemistry). (B) Photographic images of zeolite, kaolinite, cement proxy (CaO + CaCO_3_), and painted wall surfaces.

Zeolite (zeolith, Sigma) and kaolinite (natural, Sigma) thin films were prepared on 1 × 1 in. glass slides by drop-casting 50 mg of each material and kept for 24 h for slow air drying (see Figure 1A). Cement proxy films were prepared in the same manner using a mixture of 25 mg of CaO (99.95%, Alfa Aesar) and 25 mg of CaCO_3_ (99.0% calcite, Alfa Aesar). Painted wall surface films were prepared with commercially available white paint (Behr Marquee interior eggshell ultrapure white, No. 2450) applied on a wallboard block of dimension 1 × 1 in. Each painted wall sample contained ∼90 to 100 mg of paint. Representative images of the thin films are shown in Figure 1B.

### Materials Characterization

The crystalline phases of zeolite, kaolinite, CaO, and CaCO_3_ particles were confirmed with X-ray diffraction (XRD) using an APEX II ultradiffractometer with Mo Kα radiation at λ = 0.71073 Å. In this work, commercially available dehydrated zeolite A (Na_12_(AlO_2_)_12_(SiO_2_)_12_) was used, which is a small-pore zeolite consisting of an 8-ring three-dimensional cage with a charge compensating cation (Na^+^) at the center of the pore.^25^ Crystallized kaolinite particles are hexagonal platelets with one silica tetrahedral sheet and one alumina octahedral sheet held together by O–H–O bonds.^26^ CaO samples consist of a large amount of calcium hydroxide and a small amount of CaCO_3_. Calcite is used in the cement proxy sample as it is a component of limestone that is used for cement production. The surface area of these materials was determined by a 15-point N_2_-BET adsorption isotherm using a Quantachrome Nova 4200e surface area analyzer where each surface component was degassed for ∼6 h at 150 °C before the measurements. The estimated surface areas are 7.0 ± 0.7, 8.4 ± 0.5, 5.1 ± 0.5, and 7.7 ± 2.0 m^2^ g^–1^ for zeolite, kaolinite, cement, and painted wall samples. These values are averages of multiple measurements of the particles themselves before forming a film and small flakes of dried paint for the painted wall sample.

### Cavity-Enhanced UV Absorption Spectroscopy for Gas-Phase Measurements of NO_2_ and HONO

For simultaneous detection of gas-phase HONO and NO_2_, a light-emitting diode (LED)-based incoherent broadband cavity-enhanced spectrometer was used. The details of the instrumental setup have been described elsewhere.^27^ Briefly, a high-power UV LED (Nichia, NVSU333A, 3.640 W, peak wavelength λ = 365 nm) is used as the probe light source, which radiates light in the wavelength range from 360 to 390 nm, corresponding to the electronic transitions *A*^1^*A*^″^ ← *X*^1^*A*^′^ (0 – 0,1 – 0) and *A*^2^*B*_1_ ← *X*^2^*A*_1_ of HONO and NO_2_, respectively. The output from the LED was collimated using a lens assembly consisting of two aspheric condenser lenses (Thorlabs, ACL25416U-A, diameter = 1 in., NA = 0.79) and was directed into the optical cavity made of polytetrafluoroethylene (PTFE) (inner diameter = 2.54 cm) with high reflectivity mirrors (CRD Optics, 99.99% reflectivity at 370 nm, ROC = 1 meter, diameter = 2.54 cm) at each end separated by 75 cm. The current setup yields an *R*(λ) of 99.92% that leads to an average effective optical pathlength of ∼1.4 km in the wavelength range 365–390 nm.

Transmitted light exiting the cavity is collected and focused into a multimode optical fiber (Ocean optics, PL100-2-UV–VIS, diameter = 1 mm, numerical aperture (NA) = 0.22) using a plano-convex fused silica lens (Thorlabs, LA4380-UV, diameter = 1 in., antireflective (AR) coated 245–400 nm, *f*/3.93, focal length = 100 mm). Ambient scattered lights are removed using a bandpass filter (Semrock, FF01-370/36-25, 25 mm). The collected light is fed into the inlet slit (25 μm) of a fiber-coupled charge-coupled device (CCD) spectrometer (Ocean Optics, QEPro). The resulting spectral range of the CCD detector is 300–680 nm with a spectral resolution of ∼0.396 nm. The QEPro is controlled, and spectra are acquired using the OceanView software. Each spectrum is collected with an integration time of 20 s, and then, 10 spectra are averaged together. The concentrations of HONO and/or NO_2_ are extracted by performing a multivariate DOAS fit of the reference cross-sections to the acquired CEAS spectra using the DOASIS software package. This experimental setup can detect a trace amount of gas-phase HONO and NO_2_ in the 5–1000 ppb range. Heterogeneous hydrolysis of NO_2_ on particle surfaces can also result in the formation of gas-phase NO,^28,29^ which was not measured in this study. As discussed in the Supporting Information, mass balance calculations of the gas-phase products, HONO and NO_2_, and condensed-phase products, NO_2_^–^ and NO_3_^–^, measured in this study account for 80–85% of the nitrogen oxide products that form and the other 15–20% are most likely other gas-phase nitrogen oxides such as NO and N_2_O (for more details, see Figure S1 and Table S1 in the Supporting Information (SI)).

### Ion Chromatography

NO_2_-exposed samples were extracted before and after photolysis in 20 mL of deionized water and sonicated them for an hour before filtering out the suspended surface materials. Condensed-phase nitrite and nitrate were quantified using ion chromatography (IC, Dionex ICS2000) equipped with a Dionex AS25 analytical column.

### Experimental Protocols

Figure 1 summarizes several different experiments done within this study. For NO_2_ hydrolysis reaction, thin films of different building materials were exposed to a flow of a NO_2_/N_2_ gas mixture at a fixed concentration for 16 h under darkness and under illumination at a relative humidity of 45 ± 5%. As shown in Figure 1A, these experiments are referred to “dark” NO_2_ hydrolysis and “photo-enhanced” NO_2_ hydrolysis, respectively. Photo-enhanced NO_2_ hydrolysis reactions were carried out only at a NO_2_ concentration of 110 ppb. NO_2_ hydrolysis reactions in the dark were carried out at two different NO_2_ concentrations of 9 ppm (high concentration) and 110 ppb (low concentration). For nitrate (NO_3_^–^) photochemistry experiments, samples previously exposed to 9 ppm of NO_2_ for 16 h were placed in a PTFE reaction cell of 50 cm^3^ volume with a 2 in. diameter CaF_2_ window on top. A solar simulator (Newport 67005, 50–500 W) was used as the radiation source with a photon flux equivalent to 1 sun. N_2_ gas is flowed through the reaction cell at a constant rate of 100 sccm to transport the resulting gaseous products into the CEAS cavity. The relative humidity was varied in six steps in the range from 10 to 90% by changing mixing ratios between dry and the wet N_2_ gas for the RH-dependent studies. The RH is measured online during the data acquisition at a repetition rate of 0.1 Hz (Sensirion SHT85).

## Results and Discussion

### NO_2_ Hydrolysis on Different Building Materials

#### Condensed-Phase Measurements at High NO_2_ Concentrations in the Dark

The heterogeneous reaction of gas-phase NO_2_ on different surface materials under humid conditions produces HNO_3_ and HONO as shown below.^30−32^
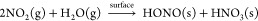
R1
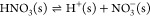
R2
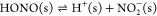
R3
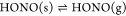
R4HNO_3_ is expected to be adsorbed on the surface as adsorbed nitrate (R2). HONO can also form condensed-phase adsorbed nitrite (R3). Alternatively, HONO can partition into the gas phase (R4). As already noted, the present study focuses on the simultaneous gas-phase and condensed-phase product measurement from heterogeneous NO_2_ hydrolysis in the light and dark as well as the photochemistry of surface adsorbed nitrate on indoor relevant model thin films composed of zeolite, kaolinite, painted wall, and the CaO + CaCO_3_ mixture as a cement proxy.

**Figure 2:**
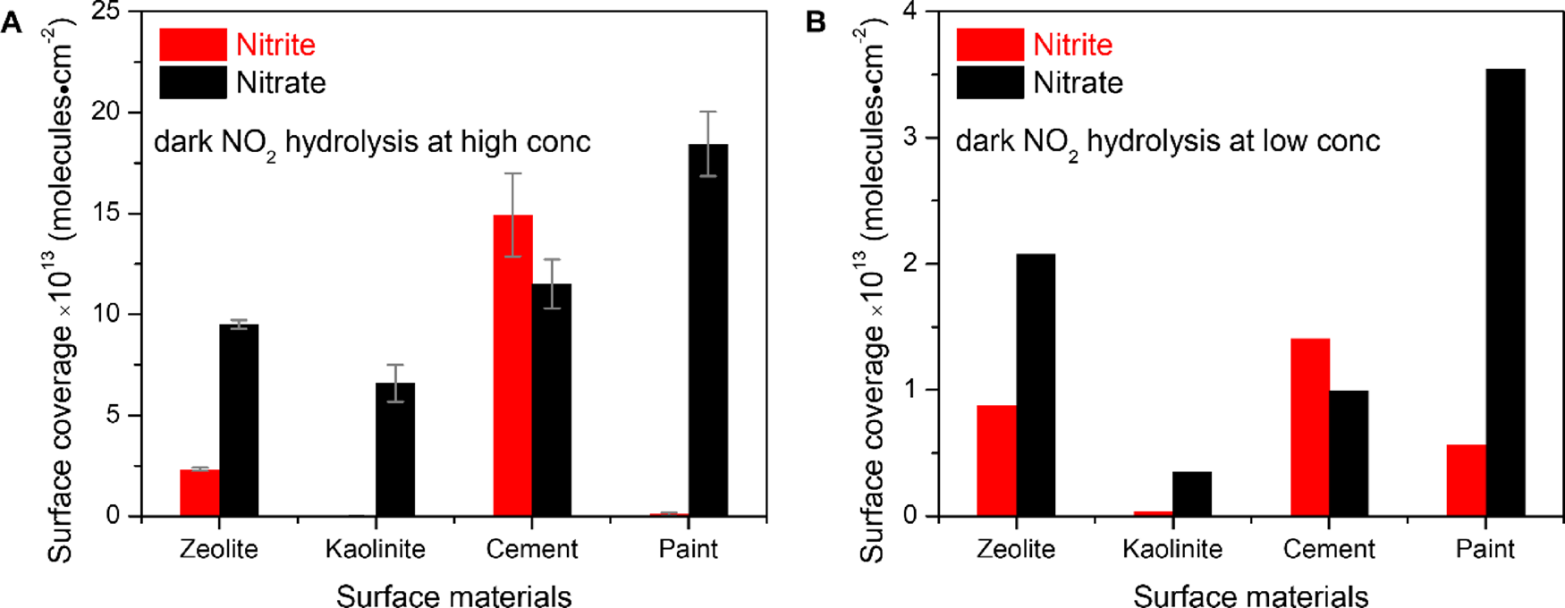
Surface coverage of nitrite (red) and nitrate (black) ions for NO_2_ uptake reaction (dark NO_2_ hydrolysis) in the dark at RH = 45 ± 5% on four different indoor material surfaces: zeolite, kaolinite, CaO + CaCO_3_ as a cement proxy, and painted wall. Thin films of different building materials were exposed to a flow of (A) 9 ppm (high conc.) and (B) 110 ppb NO_2_ (low conc.) gas mixture in N_2_ for 16 h. Data points are the average of multiple measurements, and error bars represent one sigma standard deviation uncertainties (±1σ). Note that *y*-scales are different. Surface coverages are an order of magnitude lower for NO_2_ exposure at lower concentrations compared to higher concentrations.

Condensed-phase products from dark NO_2_ hydrolysis reactions at high NO_2_ concentrations were extracted in deionized water by sonication and analyzed using ion chromatography. Figure 2A summarizes the surface compositions of four NO_2_ exposed surface materials where each sample was exposed to 9 ppm of NO_2_ for 16 h under the dark condition at RH = 45 ± 5%. Surface adsorbed nitrate was detected from all four samples with the surface coverages in the range from 0.6 to 1.8 × 10^14^ molecule cm^–2^ in the following order: paint < cement < zeolite < kaolinite. These are the average values of the multiple measurements. Surface coverage of the blank samples was in an order of ∼10^12^ molecules cm^–2^. Among the four different surface materials, only the zeolite and cement proxy samples were found to be major sinks of condensed-phase nitrite with surface nitrite coverages of 2.3 ± 0.1 × 10^13^ and 1.5 ± 0.2 × 10^14^ molecule cm^–2^, respectively. Surface nitrite concentration on the painted wall surface was just above the detection limit. No nitrite was detected on kaolinite. Surface saturation of these nitrogen oxide anions did not occur under these experimental conditions as the estimated surface coverage is smaller than the saturated surface coverage previously reported in the literature, which is in an order of ca. 5 × 10^–14^ molecules cm^–2^.^33^ In this analysis, it is being assumed that the entire sample surface area within the thin film is available for surface adsorption.

#### Condensed-Phase Measurements at Lower NO_2_ Concentrations in the Dark

In the previous section, surfaces were exposed to a NO_2_ concentration, which was higher than the average indoor NO_2_ concentration. To investigate the concentration effects, these thin films were also exposed to a flow of ∼110 ppb of NO_2_ for 16 h in the dark at RH = 45 ± 5%, where the typical indoor NO_2_ mixing ratio varies in the range from 15 to 200 ppb. Figure 2B shows the measured condensed-phase product concentration following NO_2_ exposure in the dark. Although the surface coverages were lower at low NO_2_ concentrations, all the thin films followed a similar trend at both NO_2_ concentration levels. Zeolite and cement thin films act as major HONO sinks by absorbing nitrite. A larger condensed-phase nitrite coverage was found in the NO_2_-exposed painted sample at this lower NO_2_ concentration. Most importantly, the nitrite fraction () on different surfaces was higher compared to the value at high NO_2_ concentrations. This outcome is in accord with the observation by Underwood et al. that a conversion of nitrite to nitrate occurs as surfaces are exposed longer to NO_2_.^34^

#### Gas-Phase Measurements for Photo-Enhanced NO_2_ Hydrolysis

The temporal variation of gas-phase HONO and NO_2_ concentration was also monitored before and after the introduction of the samples into the reaction cell at the lower NO_2_ concentration. The HONO concentration level was below the detection limit for the NO_2_ hydrolysis in the dark. However, the NO_2_ hydrolysis reaction was also performed in the presence of a solar simulator at 45 ± 5% RH, where gas-phase HONO was above the detection limit of this experiment for the kaolinite and painted wall thin films as these two surfaces form gas-phase nitrous acid not adsorbed nitrites. Photo-enhanced NO_2_ hydrolysis was observed for all films used in this experiment. For zeolite, the uptake coefficient was enhanced by 50%, where it was sixfold for cement and an order of magnitude higher for the painted wall and kaolinite. Some representative time traces of NO_2_ and HONO concentration are shown in Figure S1 for the photo-enhanced NO_2_ hydrolysis reaction. When the NO_2_ flow was directed over the samples, an instantaneous decrease of the initial NO_2_ mixing ratio followed by recovery was observed for all four samples. The steady-state uptake of NO_2_ was achieved at different time scales for different surfaces. For example, the steady-state uptake for the cement thin film took longer when compared to the kaolinite thin film. This result is in accord with the data presented in Figure 2B. Figure 2B shows that the coverage of nitrate + nitrite on the cement film is higher than that for kaolinite. As already noted, a mass balance analysis was performed for the photo-enhanced NO_2_ hydrolysis reaction and other gas-phase products such as NO and N_2_O make up ca. 15–20% of other nitrogen oxide products (see Section S1 and Table S1).

#### NO_2_ Hydrolysis Mechanism and the Role of Building Materials in Adsorbed Products

Previous studies of NO_2_ hydrolysis on NaY zeolite reported that under humid conditions, NO_2_ preferentially reacts with surface adsorbed water following reaction R1.^24,29,35−37^ HNO_3_ and HONO get deprotonated and stabilized by surface cationic sites forming Brønsted acidic OH groups along with surface adsorbed nitrate and nitrite according to reactions R5 and R6.^24,29,35−37^
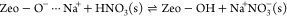
R5
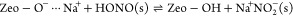
R6

In this study, zeolite is found to be a reservoir of surface nitrite. The presence of charge compensating cations and larger internal surface area can stabilize nitrites and make the gas-phase HONO production pathway through protonation of the surface nitrite unfavorable.

In a previous study, Angelini et al. predicted that the uptake reaction of NO_2_ on the kaolinite surface followed a second-order kinetic with respect to the reactive surface sites and order of 1.5 ± 0.1 with respect to NO_2_ concentration as shown in R7.^26^
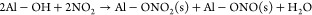
R7

The majority of the nitrate products are predicated to be associated with the octahedral aluminum hydroxide surface.^26^ No condensed-phase nitrite was detected in this study for kaolinite, which agrees with the observation by Hinrichs and co-workers, where only gas-phase HONO was detected.^26^ HONO can be released into the gas phase upon protonation of surface nitrite by the surface adsorbed water molecules (R8).^26^
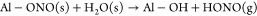
R8

This difference in nitrite-capturing ability between zeolite and kaolinite has been attributed to the microporous crystal structure and the presence of stabilizing cations (in this case Na^+^) within the zeolite pores.^24,29,35−37^

NO_2_ uptake and HNO_3_ uptake on both components of the proxy cement sample, CaO and CaCO_3_, have been studied extensively in the past. Ca(NO_3_)_2_ as surface nitrate and gas-phase NO were reported as the dominant products.^38^ In our ion chromatography experiment, an equivalent amount of surface adsorbed nitrite and nitrate were detected for the cement proxy sample. Hence, we propose the following reaction mechanism for NO_2_ uptake on the cement proxy sample under dark and humid conditions (R9R10R11R12):
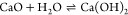
R9
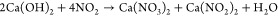
R10
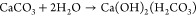
R11
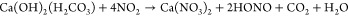
R12HNO_3_ and HONO are more likely to react with the alkaline surface materials to form calcium nitrate and calcium nitrite salt due to the basic nature of CaO and CaCO_3_. Additional experiments were carried out to investigate the surface acidity effect (vide infra).

The major components of the paint materials used in this study include titanium dioxide (10–30 w%), aluminum silicate, silica, aluminum hydroxide, and ethylene glycol (EG). TiO_2_ is a known photosensitizer, and Garcia et al. have shown enhancement of HONO production from aqueous nitrate by EG following a secondary superoxide radical mechanism.^39−44^ Under dark and humid conditions, NO_2_ uptake on TiO_2_ is expected to follow reaction R1. Previous Fourier transform infrared spectroscopy measurements for NO_2_ uptake on the TiO_2_ surface found surface nitrite as bidentate nitrito species, oxide-coordinated monodentate, bidentate, and bridging surface nitrate and gas-phase NO.^28^ In this study, the highest amount of surface adsorbed nitrate along with a small amount of surface nitrite was found for NO_2_-exposed painted film samples.

#### Role of Surface Acidity

To further examine the effect of surface acidity, the cement sample was exposed to CH_3_COOH before the NO_2_ uptake reaction. Enhancement of the gas-phase HONO level along with lower NO_2_ uptake efficiency was observed for CH_3_COOH-exposed cement samples (see Figure S1C). This suggests that surface acidity plays a role both in NO_2_ hydrolysis reaction and gas-phase HONO generation through protonation of NO_2_^–^. This surface acidity effect was further confirmed by condensed-phase nitrate and nitrite measurements from NO_2_-exposed CaO, CaCO_3,_ and, in addition, Al_2_O_3_ samples. A significant amount of nitrite was observed only on the CaO surface. Al_2_O_3_ is known as an acidic metal oxide where the p*K*_a_ values of CaO and CaCO_3_ are 12.8 and 9.0, respectively. The difference in the p*K*_a_ values could be related to this difference in reactivity. Hence, highly basic surfaces are expected to stabilize HONO as adsorbed nitrite. Hydrolysis of CaO and CaCO_3_ makes Ca(OH)_2_ (R9) and Ca(OH)_2_(H_2_CO_3_) (R11), respectively. Ca(OH)_2_(H_2_CO_3_) on the CaCO_3_ surface might provide surface sites for the protonation of nitrite to make gas-phase HONO. Therefore, it can be concluded that CaO is the important cement component for stabilizing the surface nitrites.

In summary, only zeolite and cement proxy thin films are able to store condensed-phase nitrite, which is generated from the heterogeneous hydrolysis of NO_2_. Surface adsorbed nitrate was observed in all four surface materials with different surface coverages. The nitrite to nitrate ratio was ∼1:1 in the cement proxy sample, where this ratio was ∼1:2 or ∼1:4 for zeolite depending on the NO_2_ concentration. This suggests that cement captures almost all HONO, which is being generated through NO_2_ hydrolysis. Zeolite can capture only a fraction of it. After nitrite/HONO formation, the surface composition determines the sink processes of HONO and hence the gas-phase indoor HONO mixing ratio. All four surfaces were exposed to gas-phase HONO to verify this hypothesis (see Section S2). Surface adsorbed nitrite was only detected on zeolite and cement samples (see Figure S2). As discussed previously, the presence of charge compensation cations in zeolite and the strong basicity of the cement proxy surface hinder the protonation of surface adsorbed nitrite to form gas-phase HONO.

### Broad Solar Irradiation of NO_2_-Exposed Surfaces under Humid Conditions

#### Gas-Phase Measurements with Light and Varying Relative Humidity

The reactive uptake and hydrolysis of NO_2_ in the dark (dark NO_2_ hydrolysis at high concentrations) yield surface adsorbed nitrate and/or nitrite as discussed above. The hydrolysis of NO_2_ under simulated solar irradiation shows that these reactions are enhanced, as discussed above. In order to better understand this photo-enhancement and the potential role of nitrate photochemistry, different building materials were first exposed to NO_2_ in the dark at high concentrations (9 ppm). The flow of NO_2_ was then turned off, and the different thin film samples, which now contain nitrate and/or nitrite, were then irradiated with broadband solar light. Gas-phase HONO and NO_2_ products were measured from photochemistry under broadband illumination as a function of RH. Nitrate photochemistry has been shown to lead to HONO and NO_2_; this was further explored as discussed below.

**Figure 3:**
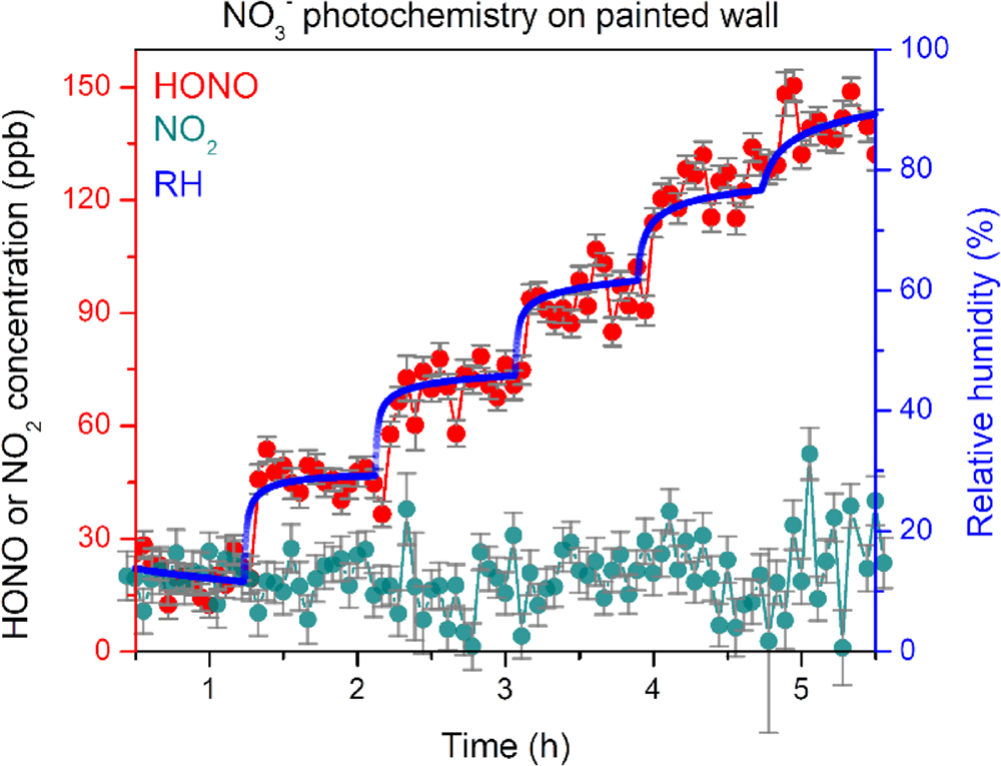
RH-dependent gas-phase HONO (red) and NO_2_ (cyan) concentrations generated from NO_3_^–^ photochemistry on the painted wall surface, which had been exposed to 9 ppm of NO_2_ exposed in the dark for 16 h and subsequently irradiated with a solar simulator. RH is varied in six steps as indicated by the blue lines. Spectra are recorded every 200 s, and the system was allowed to equilibrate at each RH before moving to the next RH. At a 100 sccm flow rate, signal intensity within our spectrometer takes ca. 30 min to equilibrate. Here, error bars represent uncertainties (±1σ) from individual DOAS fitting, which is found to be the largest source of error in this experiment. RH values have an uncertainty of ±5%. Due to gas-phase photolysis, there is an estimated depletion of <10% HONO and <5% NO_2_ from the gas phase.

Figure 3 depicts typical gas-phase HONO and NO_2_ concentrations from the NO_2_-exposed painted surface under light irradiation where the RH is varied in six steps in the range from 10 to 90%. Under each RH condition, the gas-phase product signal intensities were allowed to equilibrate. Triplicate measurements were conducted for each sample under each RH condition. We have previously shown that photolysis is responsible for <10% and <5% gas-phase HONO and NO_2_ loss, respectively.^27^ The product concentration reduces over time due to the loss of surface nitrate. As a result, a time varied correction factor is applied to compensate it as discussed in more detail in the SI (see Section S3 and Figure S3). Additionally, background HONO and NO_2_ concentrations from NO_2_-exposed thin films in the dark and under humid conditions, along with condensed-phase nitrate and nitrite, were also measured (see Section S4, Figures S4 and S5). The enhancement in the gas-phase products from nitrate photochemistry was then determined as discussed below.

**Figure 4:**
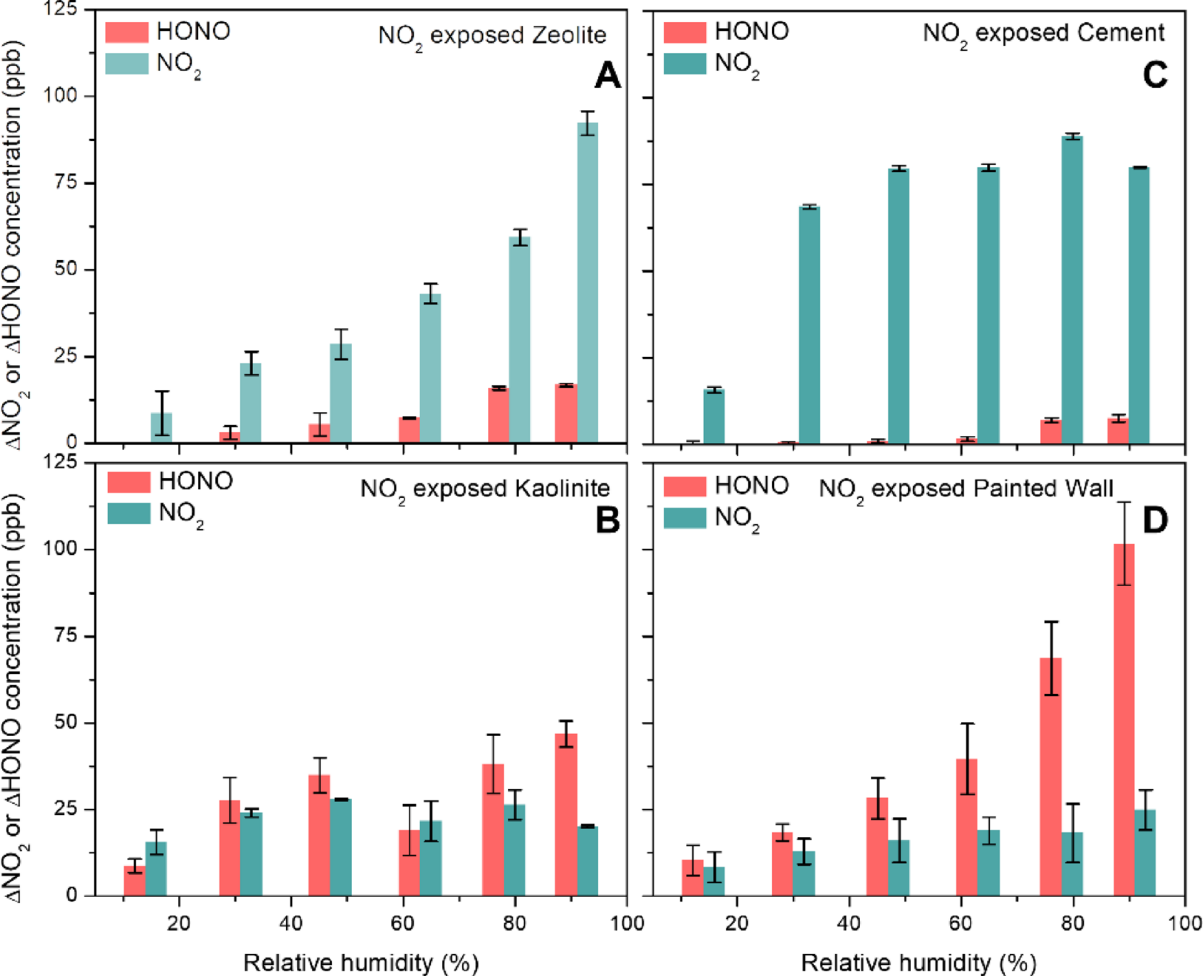
Gas-phase HONO (red) and NO_2_ (cyan) concentration from NO_3_^–^ photochemistry as a function of relative humidity in the presence of solar irradiation of surface films: (A) zeolite, (B) kaolinite, (C) CaO + CaCO_3_ mixture as cement proxy, and (D) painted wall that had been exposed to 9 ppm of NO_2_ for 16 h. Background HONO and NO_2_ concentrations under darkness but humid conditions were subtracted so as to determine the net photo-enhanced concentrations of gas-phase products, i.e., ΔNO_2_ and ΔHONO. Data points are the average of triplicate measurements, and error bars represent uncertainties (±1σ).

The photochemistry of surface adsorbed nitrate has been studied, and it is known to produce gas-phase HONO and NO_2_.^27,45^ Aqueous nitrate ions absorb lights in the 200–400 nm wavelength region corresponding to an intense π → π* transition around 200 nm and an *n* → π* transition peaking near 310 nm.^30^ The *n* → π* bands for surface adsorbed nitrates are expected to be red-shifted and fall into the spectral irradiance of the sunlight.^45,46^ Previous studies have discussed an enhancement of absorption cross-sections of surface adsorbed nitrate to be 3–4 orders of magnitude higher compared to aqueous nitrate or gas-phase HNO_3_ in the wavelength range > 310 nm.^47−50^ As a result, the experimental photolysis rate constant of surface adsorbed nitrate may be 2–3 orders of magnitude higher than the photolysis rate constant in the solution or in the gas phase.^51^ Numerous studies have shown that photolysis of nitrate predominantly produces NO_2_ and NO_2_^–^ (R13A and R13B). Protonation of NO_2_^–^ (R14) and heterogeneous hydrolysis of NO_2_ (R1) can produce gas-phase HONO.^30,46,52^
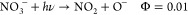
R13A
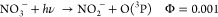
R13B
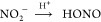
R14Figure 4 shows the HONO and NO_2_ concentrations measured from irradiated samples that had been previously exposed to NO_2_ as a function of RH. This difference ΔHONO and ΔNO_2_ is obtained by subtracting the gas-phase concentration in the dark from the concentration under illumination (see Figure S6) at the same RH. The effect of solar photon flux in the renoxification process was evident. Both the gas-phase products HONO and NO_2_ were observed for all four surfaces for all RH conditions.

As shown in Figure 4, the photo-enhanced gas-phase HONO concentration increases gradually with the increase of RH for zeolite, cement proxy, and painted wall surfaces. Except for zeolite, the fraction of photo-enhanced HONO concentration increases with the increase of RH. The presence of surface adsorbed water can enhance proton mobility and acidity of the surface, which can result in acceleration of nitrite protonation or NO_2_ hydrolysis and therefore the enhancement of gas-phase HONO production. Surface-specific relative humidity effects are discussed in the SI (Section S5). Like HONO, the NO_2_ concentration increases with the increase of RH for zeolite, cement proxy, and painted wall surfaces in the presence of light. This suggests that adsorbed water facilitates photolysis reaction R13A to form NO_2_.

#### Role of Surface Material Composition in Nitrate Photochemistry

Gas-phase product concentrations from nitrate photochemistry are significantly different on the different surfaces. The painted wall and kaolinite can efficiently convert surface adsorbed nitrate to HONO under illumination and humid conditions. On the contrary, NO_2_ is the major photoproduct for zeolite and cement proxy. A similar trend was observed even in the dark conditions (see Figures S4 and S6). Most strikingly, only a small amount of HONO was generated from the zeolite and cement proxy surfaces where substantially large amounts of surface adsorbed nitrites are stored. These surface adsorbed nitrites do not readily get protonated to form gas-phase HONO even at a very high RH. In contrast, a notable amount of HONO signal was detected when there was a minimal amount of surface nitrite on kaolinite and painted wall surfaces. Zeolite and cement are good sinks of HONO, whereas painted walls and kaolinite are efficient sources of HONO. This implies that the surface interaction of adsorbed nitrite is the dominant factor controlling the gas-phase HONO mixing ratio in an indoor air environment.

The maximum amount of gas-phase HONO generated from the painted surface (15–100 ppb) is followed by the kaolinite surface (15–60 ppb). It is well established that photoexcitation of TiO_2_ < 390 nm forms electrons in the conduction band and holes in the valance band. Electrons reduce NO_2_ to nitrite where holes oxidize water to form OH radicals and protons. Subsequent protonation of nitrite would make HONO as depicted in reaction R15R16R17.^40^
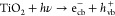
R15
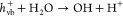
R16
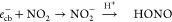
R17

Additionally, EG, present in the painted sample, can act as a OH scavenger and can enhance the nitrite yield from nitrate photolysis.

There is a stark difference between the two aluminosilicate minerals: zeolite and kaolinite. It can be argued that the charge compensating cation in a confined space in zeolite stabilizes photolytically generated nitrite and hinders surface adsorbed water to protonate nitrite to form gas-phase HONO. Amphoteric aluminum hydroxide in kaolinite cannot stabilize nitrite in the condensed phase and allows the release of gas-phase HONO like hydrated silica or Al_2_O_3_.^38,53^

The lowest gas-phase HONO percentage from the alkaline cement surface suggests that surface acidity/basicity might play an important role in determining the gas-phase HONO mixing ratio as discussed in the previous section. This result complements the observations by Abbatt and co-workers during the HOMEChem campaign (2018); house floor mopping with vinegar solutions enhances the gas-phase mixing ratio of HONO.^54^ This study provides direct evidence of some of the mechanisms suggested by Abbatt and co-workers.^54^ Alkaline surface materials such as grout and concrete are found to be a good reservoir of nitrite, and vinegar solution could alter the surface pH to facilitate the protonation step of reaction R14 or the protonation of Ca(NO_2_)_2_.

In summary, nitrate photolysis predominantly forms HONO on kaolinite and painted wall surfaces and NO_2_ on zeolite and cement surfaces under humid conditions. This implies that good nitrite reservoirs such as zeolite and cement proxy are not good gas-phase HONO sources even at a high relative humidity. On contrary, surfaces like kaolinite and painted surfaces are good sources of HONO like Al_2_O_3_ and TiO_2_.^38,53^

#### Condensed-Phase Measurements Following Broadband Irradiation

Measurements of the surface coverage of nitrate and nitrite were also performed on the same NO_2_ exposed surfaces at the end of the gas-phase photolysis experiments (see Figure S7). Surface adsorbed nitrate loss was observed for the zeolite, kaolinite, and painted surfaces along with a small growth of nitrite coverage for zeolite. A drastically different result was found for the cement proxy surface: loss of nitrite coverage and rise of nitrate coverage. Detailed discussion is presented in the SI (see Section S6). In summary, photolysis of nitrite leads to O^–^ that then oxidizes nitrite to nitrate.

### Implications of Material Specific HONO Chemistry in Indoor Environments

A systematic investigation was carried out to explore the roles of relative humidity, solar light, and specific surface properties such as surface acidity. Some of the key information is summarized in Table 1. The findings of this study indicate that HONO generation from NO_2_ uptake reaction or the photochemistry of surface deposited nitrate strongly depends on the surface materials. Zeolite and cement are condensed-phase nitrite reservoirs that do not release significant amounts of HONO through protonation of surface nitrite even at high RH or when irradiated. NO_2_-exposed kaolinite and painted surfaces readily release gas-phase HONO under humid conditions, which is enhanced significantly upon irradiation. Like NO_2_-exposed surfaces, condensed-phase nitrite was only observed in HONO-exposed zeolite and cement proxy samples.

**Table 1:** Summary of the Primary Condensed Phase from NO_2_ Hydrolysis Reaction at High Concentrations and the Subsequent Gas-Phase Products from NO_3_^–^ Photochemistry^a^

material surfaces	NO_2_ hydrolysis (high concentration)^b^	NO_3_^–^ photochemistry
	condensed-phase products	major gas-phase product
painted wall	NO_3_^–^	HONO
kaolinite	NO_3_^–^	HONO
zeolite	NO_3_^–^ and NO_2_^–^	NO_2_
cement	NO_3_^–^ and NO_2_^–^	NO_2_

a Building materials that store NO_2_^–^ produce NO_2_ as the major gas-phase product and not HONO.

b NO_2_ hydrolysis under lower concentrations showed minor nitrite production on kaolinite and painted surfaces compared to nitrate.

**Figure 5:**
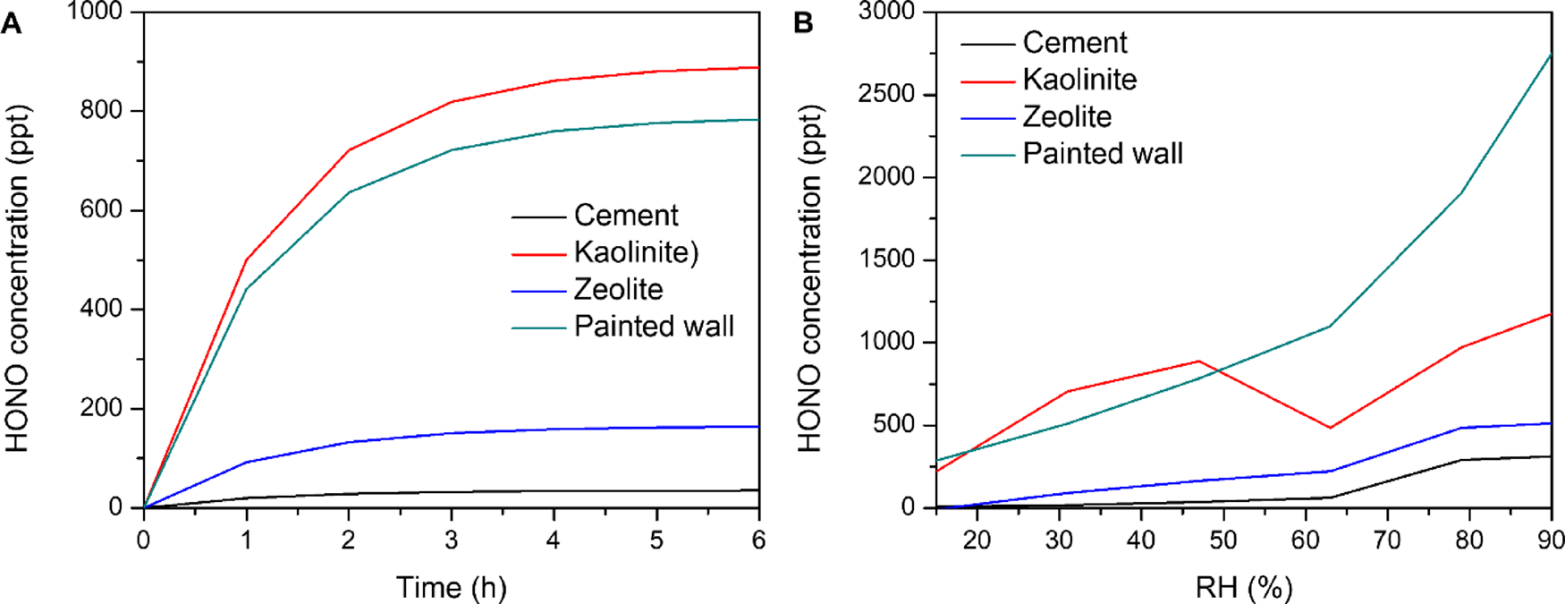
(A) Estimated (eq S3) HONO concentration from nitrate photochemistry on cement (black), kaolinite (red), zeolite (blue), and painted wall (cyan) surfaces in an indoor air environment when one-fifth of the indoor volume is directly illuminated by sun light and at RH = 45 ± 5%. (B) RH-dependent HONO mixing ratios for all four samples after 6 h of NO_3_^–^ photochemistry under similar lighting conditions.

Heterogeneous reaction of NO_2_ on the particle surface results in the formation of NO, which was not measured in this study. A mass balance approach is used in the SI (Section S1 and Table S1) to estimate the NO and other NO_*x*_ products. Based on this, we estimate that approximately 15–20% products from NO_2_ uptake under solar illumination are not HONO or NO_2_ but in fact other gas-phase nitrogen oxides such as NO and N_2_O. Based on the experimental outcome, HONO mixing ratios from nitrate photochemistry in a realistic indoor environment were simulated where the details can be found in the SI (Section S7). The results are presented in Figure 5. HONO concentration is predicted for four different samples (Figure 5A) at an indoor relevant humidity RH = 45 ± 5%. The painted wall and kaolinite produce an order of magnitude more HONO from nitrate photochemistry compared to cement and zeolite surfaces. RH dependence of HONO formation on kaolinite and painted surfaces is calculated and shown in Figure 5B in the RH range from 15 to 90% after 6 h of reactions. HONO mixing ratios do not change significantly with RH for kaolinite. However, the simulation predicts that the HONO mixing ratio on painted wall surfaces can elevate from ∼1 ppb at RH = 45% to 3 ppb at RH = 90%. In data analysis and in the simulation, it is assumed that the entire surface materials were involved in the reaction. However, this may not be true, and to address, thus, HONO estimation for a typical room under indoor relevant conditions, we considered 100, 50, and 30% of surface materials available for reaction (see Figure S8).

Hence, some building materials are more efficient as condense-phase nitrite reservoirs, whereas some building materials are efficient sources of gas-phase HONO. The main conclusion of this study suggests that the indoor HONO mixing ratio is strongly controlled by the surface material. Overall, indoor heterogeneous nitrogen oxide chemistry is highly material surface specific. The type of building material, surface composition, and surface acidity all play a role in determining the indoor HONO budget. Any indoor chemistry model should include specific information about surface composition along with other factors such as temperature, photon flux, and RH to correctly predict indoor HONO chemistry.

## Supporting Information Available

Time course experiments for photo-enhanced NO_2_ hydrolysis; surface coverages of condensed-phase nitrate and nitrite for photo-enhanced NO_2_ uptake; condensed-phase nitrite coverages following HONO uptake on thin-film surfaces; decrease in gas-phase HONO concentrations over time due to gas-phase HONO photochemistry; HONO and NO_2_ formation in the dark from surface adsorbed nitrate and nitrite under humid conditions; changes in nitrite and nitrate concentrations in the dark following increasing relative humidity; photo-enhanced HONO production from nitrate photochemistry as a function of relative humidity; photo-enhanced changes in nitrite and nitrate concentrations with increasing relative humidity; and HONO concentrations in indoor environments from nitrate photochemistry (PDF)
