# Supplementary material for: Gas-Phase Nitrous Acid (HONO) Is Controlled by Surface Interactions of Adsorbed Nitrite (NO2–) on Common Indoor Material Surfaces
Source: Environ Sci Technol. 2022 Aug 24;56(17):12045–54. doi: 10.1021/acs.est.2c02042 (PMC9454260; doi:10.1021/acs.est.2c02042)
Supplement: Supplementary file 1 — es2c02042_si_001.pdf [file es2c02042_si_001.pdf]

Supporting Information:

Gas-Phase Nitrous Acid (HONO) Formation Is Controlled by Surface Interactions of Adsorbed Nitrite (NO_2^-) on Common Indoor Material Surfaces

Shubhrangshu Pandit and Vicki H. Grassian*

Department of Chemistry and Biochemistry, University of California San Diego, La Jolla, CA 92093, USA

*Email: vhgrassian@ucsd.edu

The supporting information document contains **8 figures, 1 table,** and **14 pages** in total.

S.1. Mass balance for photo-enhanced NO_2 hydrolysis

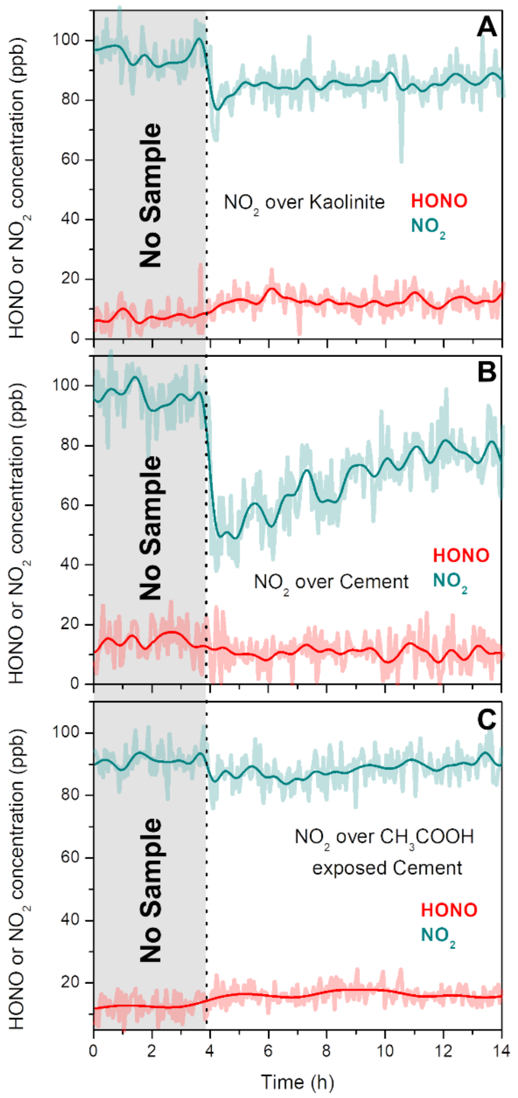

Figure S1. Change in gas-phase HONO or NO_2 concentration from photo-enhanced NO_2 hydrolysis reaction in presence of A) kaolinite, B) cement, and C) CH_3COOH exposed cement samples. Thin films were exposed to a fixed quantity of NO_2 (~110 ppb) at 45±5 % RH in the presence of solar light. For kaolinite, NO_2 level depleted by ~15 ppb and approximately makes 5 ppb of gas-phase HONO under steady -state condition. For the cement sample, gas-phase HONO concentrations were below the background HONO mixing ratio.

Along with the condensed-phase products, gas-phase HONO production and NO_2 loss were monitored during surface exposure at lower NO_2 concentration (~110 ppb) in the presence of solar light at 45 ± 5 % RH. Typical uptake profiles of NO_2 on kaolinite and cement surfaces and

subsequent HONO formation under illumination are presented in Figure S1. A large initial uptake of NO_2 was observed that slowly reaches steady-state condition. Heterogeneous reaction of NO_2 on particle surface results in the formation of NO, which was not probed in this study. A total of $3.9 \pm 0.5 \times 10^{16}$ molecules and $9.9 \pm 0.5 \times 10^{16}$ molecules of NO_2 were adsorbed by the kaolinite and cement surfaces, respectively over a period of 16 h. Steady state concentration of HONO was ~5 ppb above the background signal for kaolinite surface which is equivalent to 1.3×10^{16} molecules. HONO concentrations from the cement surface film was below the signal to noise ratio. Condensed-phase nitrate and nitrite concentrations are summarized in Table S1. Taking all of this into account, we estimate that approximately 15-20% of products are other nitrogen-containing products, most likely other gas-phase nitrogen oxides such as NO or N_2O.

Table S1. Mass balance for photo-enhanced NO_2 uptake reaction on kaolinite and cement at lower NO_2 concentration

	**Total number of molecules or ions × 10^{16}**		
	**Gas-phase**		**Condensed-phase**
**Surfaces**	**NO_2 loss**	**HONO**	**Nitrate + Nitrite**
Kaolinite	3.9 ± 0.5	1.3 ± 0.2	1.8 ± 0.1
Cement	9.9 ± 0.5	-	8.4 ± 0.1

S.2. HONO uptake

There are some direct sources of indoor HONO such as gas stove cooking, space heaters usage, and candle burning beside the conversion of NO_2 to HONO. Additionally, Collins et al have suggested that the gas-surface equilibrium of HONO controls the indoor HONO level. Hence, all these surfaces were exposed to ~200 ppb of HONO under the dark condition at RH = 45±5 % to determine their efficiency as a HONO reservoir. Figure S2 shows the condensed-phase nitrite coverage following HONO exposure for 16 h. Like NO_2 exposed samples, nitrite concentration

was significant on zeolite and cement proxy surfaces. This result further supports the conclusions that only some surfaces act as nitrite reservoirs.

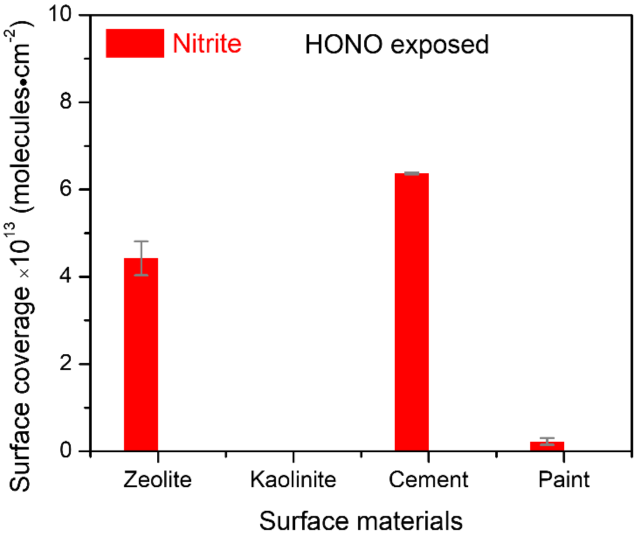

Figure S2. Surface coverage of nitrite ions on four different indoor model surfaces zeolite, kaolinite, CaO + $CaCO_3$ as cement proxy, and painted wall following exposure to ~200 ppb of HONO for 16 h in the dark and at RH = 45±5 %. Data points are the average of multiple measurements, and error bars represent uncertainties of ±1σ.

S.3. The effect of surface nitrate loss over time

HONO production should be a function of the concentration of surface adsorbed nitrate or nitrite unless it is present in large excess. The effect of surface nitrite or nitrate loss on gas-phase HONO concentration was examined by photolyzing NO_2 exposed surface for a long time at a fixed reaction condition; solar radiation at RH = 45±5 %. As shown in Figure S3, HONO concentration dropped over time for NO_2 exposed painted surface. Gas-phase HONO concentration ($[HONO]_{measured}$) was fitted to an exponential decay function eq. S1.

$$[HONO]_{measured} = A * e^{-kt} \qquad Eq. S1$$

Here parameter A is a proportionality constant, t = time, and k = decay constant. Fitted decay constants for different surfaces are determined to be 0.15+0.01 s^{-1} for these surfaces. An average of 15-16% correction in concentration was required due to surface adsorbed nitrate loss over time. The effect of surface nitrate and nitrite loss was countered using eq. S2.

$$[HONO]_{corrected} = [HONO]_{measured} * e^{kt} \qquad Eq. S2$$

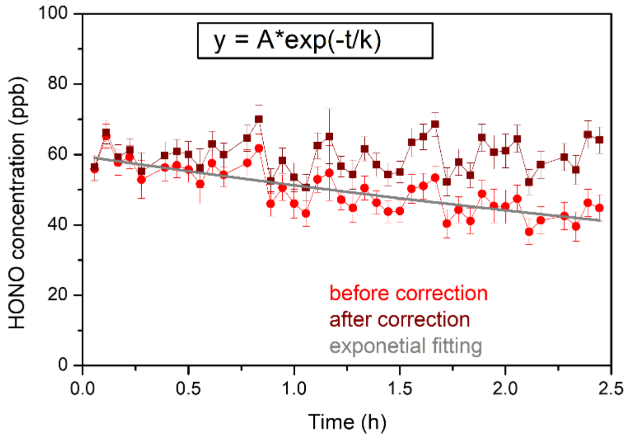

Figure S3. Time variation of gas-phase HONO concentration measured (red) from NO_2 exposed painted surface under solar illumination at RH = 45±5 %. Greyline represent an exponential fit to equation S1 of the experimentally measured HONO concentration ($[HONO]_{measured}$) shown in red colored circles. Corrected HONO concentration ($[HONO]_{corrected}$), obtained using eq. S2, is shown in the wine colored square data points in the figure.

S.4. HONO generation in the dark from surface adsorbed nitrate under humid conditions

RH-dependent HONO production from surface adsorbed nitrate and nitrite was also performed. Figure S4 presents the RH-dependent HONO and NO_2 concentrations produced from NO_2 exposed surfaces under dark conditions. These concentrations are the average of multiple measurements. Gas-phase product concentrations are significant differences on different surfaces. NO_2 is the dominant product from zeolite and cement proxy samples where kaolinite and painted walls

primarily produce HONO. The maximum amount of gas-phase HONO is being generated on the painted surface (10-60 ppb) followed by the kaolinite surface (5-45 ppb). Gas-phase HONO is the major product for these two samples where the HONO mixing ratio increases with the increase of the RH except for the highest RH value. This trend suggests that the adsorbed water plays an important role in the renoxification reaction to produce HONO from NO_2 exposed painted walls and kaolinite surfaces. RH-dependent HONO concentration from the cement proxy sample follows the same trend but the overall HONO concentration is significantly lower (5-10 ppb) than the other two surfaces. Similar to the cement sample, a smaller amount (5-15 ppb) of HONO was produced from the zeolite surface which, follows a different RH dependence; an enhancement up to RH = 30% and drops after that. NO_2 is the dominant product for zeolite and cement proxy where its concentration remained the same at all RH. This implies NO_2 degassing from these surfaces takes place at a fixed rate which is independent of RH. However, a gradual decrease in NO_2 concentration was observed for the painted wall surface. This could imply that surface adsorbed water enhances NO_2 hydrolysis to increase gas-phase HONO concentrations. RH-dependent NO_2 formation on kaolinite follows a complex trend; initial increase up to RH=30% followed by a dip and a small enhancement >60% RH. A combination of two processes might be occurring simultaneously a) replacement of adsorbed NO_2 or N_2O_4 by water molecules and b) increased water adsorption opens up more reactive sites as previously discussed by Liu et al.[2]

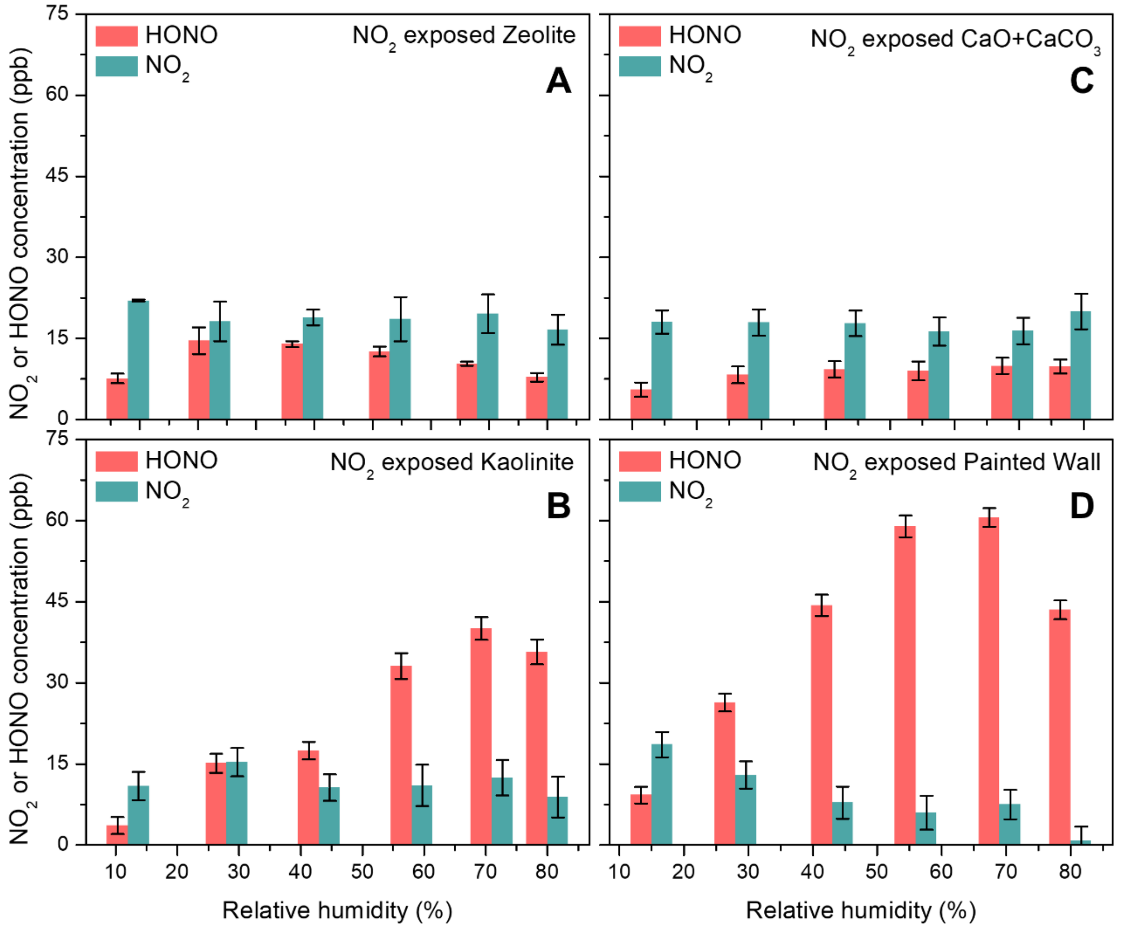

Figure S4. Gas-phase HONO (red) and NO_2 (cyan) concentration under dark as a function of relative humidity for NO_2–exposed samples: (A) zeolite, (B) kaolinite, (C) CaO + $CaCO_3$ mixture as cement proxy, and (D) painted wall. Data points are the average of multiple measurements, and error bars represent uncertainties (±1σ).

Most strikingly, only a smaller amount of HONO was generated under dark conditions from zeolite and cement proxy surfaces where substantially large amounts of surface adsorbed nitrites are stored. These surface adsorbed nitrites do not readily get protonated to make gas-phase HONO even at a very high RH. In contrast, a notable amount of HONO signal was detected when there was a minimal amount of surface nitrite on kaolinite and painted wall surfaces. This result complements the observations by Abbatt co-workers during the HOMEChem campaign (2018); house floor mopping with vinegar solutions enhances the gas-phase mixing ratio of HONO.[3]

Alkaline surface materials such as grout and concrete are found to be a good reservoir of nitrite and vinegar solution could alter the surface pH to facilitate the protonation step of reaction RS1 and RS2 or the protonation of $Ca(NO_2)_2$. Hence, we propose the following reaction mechanism for HONO formation in the dark:[4]

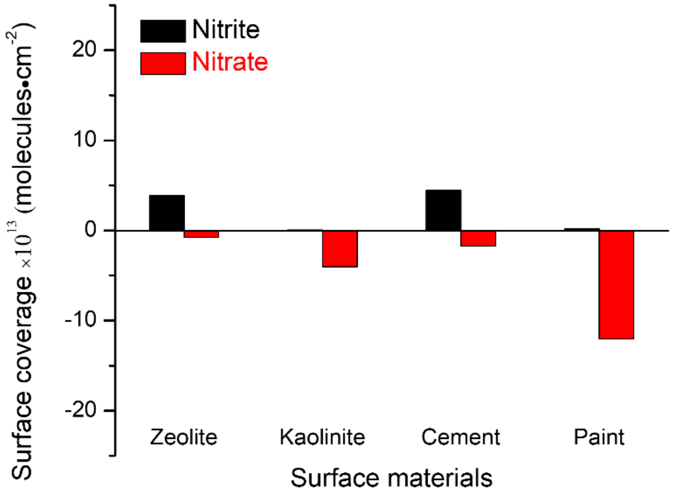

Figure S5. Change in surface coverage of nitrite (black) and nitrate (red) ions for NO_2 exposed surfaces after being introduced to RH for 6 h. A negative value represents the depletion, and a positive value signifies growth. For all four surfaces, nitrate was depleted where nitrate ion concentration was enhanced.

$$MO^- + NO_2(g) \rightleftharpoons MO - NO_2^-(s) \xrightarrow{+H_2O} MO - OH + HONO(g) \qquad RS1$$

$$MO + 2NO_2 \rightleftharpoons MO - N_2O_4(s)$$

$$\xleftrightarrow{isomerization} MO - ONONO_2(s) \rightleftharpoons MO - NO^+NO_3^-(s) \xrightarrow{+H_2O} HONO(g) + MO - NO_3^- \cdots H^+(s) \qquad RS2$$

Condensed-phase nitrate and nitrite concentration were measured after humidity treatment for 6 h. Figure S5 presents the change in surface coverage of nitrite and nitrate ions after humidity treatment. Surface coverage of nitrate was depleted for all four surfaces to make gas-phase HONO

and NO_2. An enhancement of nitrite concentration was observed for zeolite and cement samples. HONO generated through reactions RS1 and RS2 could be stabilized as nitrite on the surfaces.

S.5. Photo-enhanced gas-phase HONO production

Changes in the HONO concentration as a function of RH for NO_2 exposed samples in the presence of solar radiation are shown in Figure S6. Total HONO and NO_2 concentrations are the sum of products from two different mechanism: some produced in the dark as explained in the previous sections (sec. S.4) and some from nitrate photochemistry. Figure 4 in the main text presents the photo-enhanced fraction (ΔHONO and ΔNO_2) of the gas-phase products. Photo-enhanced HONO concentration increases with the increase of RH as surface adsorbed water facilitates NO_2 hydrolysis or protonation of nitrite. It is to point out that multilayer water uptake or liquification on the surface can hinder the water-to-air exchange rate of HONO as previously seen on gypsum surfaces.[1] Additionally, a greater wall loss of highly water-soluble HONO can reduce the gas-phase photo products. For zeolite, cement proxy, and painted wall, enhancement is higher than suppression. Kaolinite surface follows a complex trend; initial increase up to RH=45% followed by a dip and a small enhancement >70% RH. This could be the result of two compensating factors that are operating in kaolinite as discussed in the previous section. A combination of two processes might be occurring simultaneously as previously discussed by Liu et al. a) reduction of gas-surface exchange due to surface adsorbed water molecules and b) increased water adsorption opens up more reactive sites.[2] For the cement material, HONO percentage increases with RH except at the lowest RH condition. This can be argued that the surface −OH determines the reaction rate of NO_2 hydrolysis at dry conditions. As RH increases, water starts to condense on the surface and makes NO_2 hydrolysis more feasible in the liquid phase. Additionally, NO_2 hydrolysis at the photoactive

site makes HNO_3 which can change the pH of the surface locally and can enhance the protonation of nitrite.

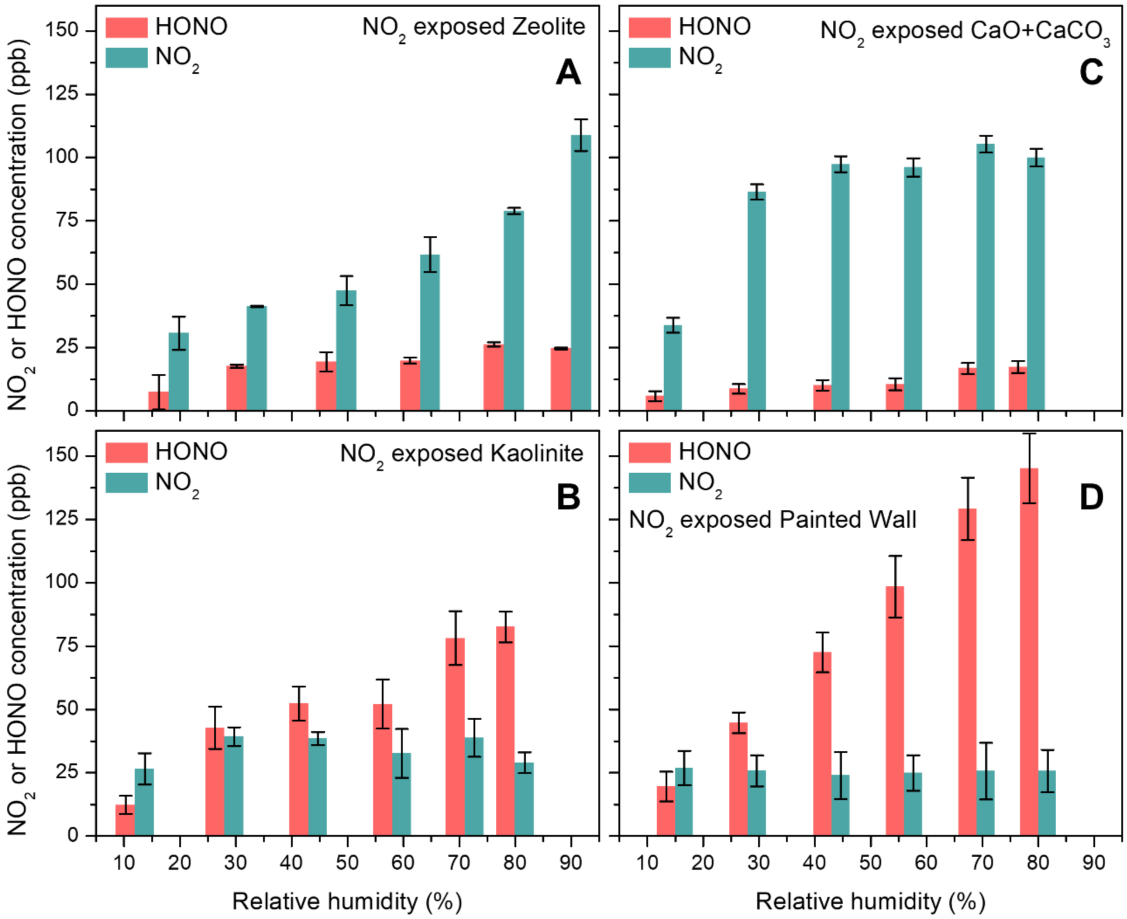

Figure S6. Gas-phase HONO (red) and NO_2 (cyan) concentrations as a function of RH for solar-irradiated NO_2–exposed thin films (A) zeolite, (B) kaolinite, (C) CaO + $CaCO_3$ as cement proxy, and (D) painted wall in the presence of solar simulator. Data points are the average of triplicate measurements, and error bars represents a sigma uncertainty.

S.6. Condensed-phase measurements of NO_2 exposed samples after broadband irradiation.

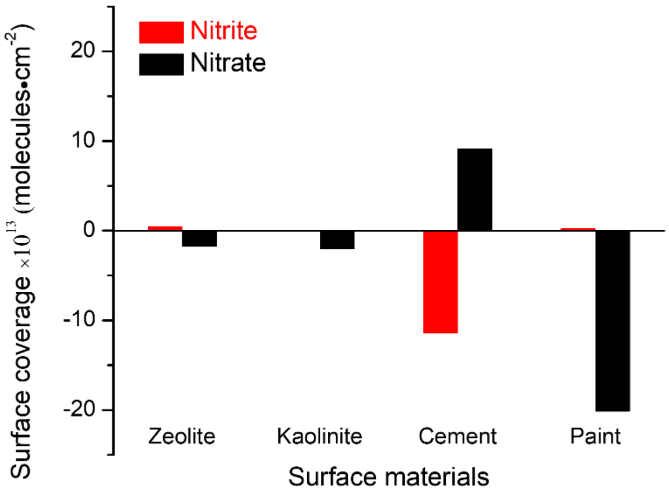

Figure S7. Change in surface coverage of nitrite (red) and nitrate (black) ions for NO_2 exposed surfaces after broadband irradiation for 6 h under humid conditions. A negative value represents the depletion of nitrite or nitrate. A positive value signifies growth.

Measurements of the surface coverage of nitrate and nitrite were also performed on the same NO_2 exposed surfaces at the end of the gas-phase photolysis experiments. Figure S7 presents the change in surface coverage by subtracting the surface coverage measured using ion chromatography before and after photolysis where each sample was exposed to light and humidity for 6 h. A negative value represents the loss of surface coverage. Nitrate loss is apparent for zeolite, kaolinite, and painted surface. For zeolite and painted surfaces, nitrate concentration reduces upon irradiation along with a small growth of nitrite coverage. Nitrate photolysis under humid conditions makes nitrite which can be stabilized by zeolite or porous painted surface. Therefore, the loss of nitrate resulted in the growth of nitrite concentration. Alternatively, nitrate can directly convert to nitrite; photoisomerization to peroxynitrite followed by rapid dissociation.[5]

$$NO_3^-(s) \overset{h\nu}{\leftrightarrow} ONOO^-(s) \overset{h\nu}{\rightarrow} NO_2^-(s) + O(^3P) \qquad RS3$$

A drastically different result was found for the cement proxy surface; loss of nitrite coverage and rise of nitrate coverage. This implies that some of the nitrites were converted to nitrate upon photolysis. Here we propose the following pathway which involves the photolysis of nitrite. Nitrite has three distinct UV absorption bands: a π → π* transition around 220 nm and two bands peaking near 318 nm and 354 nm, corresponding to n → π* transition.[6]

$$NO_2^- + h\nu \rightarrow NO + O^- \qquad RS4$$

$$3NO \rightarrow N_2O + NO_2 \qquad RS5$$

$$NO_2 + O^- \rightarrow NO_3^- \qquad RS6$$

$$NO_2^- + O(^3P) \rightarrow NO_3^- \qquad RS7$$

Nitrite photochemistry makes NO which could lead to the formation of N_2O and NO_2 in a secondary reaction. Although NO and N_2O were not probed in this work, previous nitrate photolysis studies reported N_2O and NO as gas-phase photoproducts. Alternatively, nitrite can be directly converted to nitrate by the oxygen atom generated in reaction R16B. Most importantly, nitrite to nitrate conversion upon illumination is significant on the alkaline cement surface. Thus, photochemistry under humid condition is complex and associated with loss of nitrate and nitrite along with nitrite to nitrate conversion.

S.7. Implications for indoor air environments

To estimate the concentration of HONO resulting from irradiated kaolinite clay surfaces, following equation can be used.[7]

$$C_{HONO}(t) = \frac{E_{HONO} \times S}{k_{AER}V + J(HONO)V_1}\left[1 - e^{-(k_{AER} + J(HONO))t}\right] \qquad Eq.S3$$

where $C_{HONO}(t)$ is the gas-phase HONO concentration (mg m^{-3}) in a room, E_{HONO} is the emission rate of HONO in mg m^{-2} h^{-1}, S is the surface area of the room (m^2), V is the room volume (m^3), k_{AER} is the air exchange rate (h^{-1}), and $J(HONO)$ is the photolysis rate of indoor HONO. It is worth mentioning that outdoor HONO levels are usually lower than indoor levels and it assumed to be zero in the above expression.

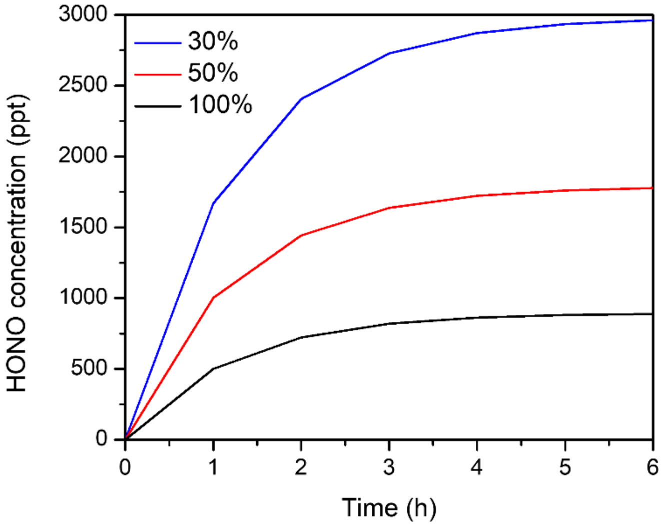

Figure S8. The estimated (Eq. S3) HONO concentration from NO_3^- photochemistry on kaolinite surface in an indoor air environment when one-fifth of the indoor volume is directly illuminated by sun light and at RH = 45 ± 5 %.. HONO concentration is calculated considering 100% (black), 50% (red), and 30% (blue) of surface materials available for reaction.

Consider an unfurnished room with a total volume of V = 50 m^3, total surface area of 85 m^2 (S/V = 1.7 m^{-1}), and total wall and ceiling area made of kaolinite S = 55 m^2. Assuming only one fifth of the room volume is irradiated by direct solar light V_1 = 10 m^3, an average air exchange rate k_{AER} = 0.56 h^{-1}, and HONO photolysis rate $J(HONO)$ = 0.26 h^{-1}, HONO concentration is predicted in Figure 5 in the main text. In this calculation and in the condensed-phase data analysis, it is assumed that the entire surface materials were involved in the reaction. Indoor HONO concentration is

calculated considering 100%, 50%, and 30% of surface materials being reacted for kaolinite sample.

REFERENCES

(1) Pandit, S.; Mora Garcia, S. L.; Grassian, V. H. HONO Production from Gypsum Surfaces Following Exposure to NO 2 and HNO 3 : Roles of Relative Humidity and Light Source. *Environ. Sci. Technol.* **2021**, *55* (14), 9761–9772. https://doi.org/10.1021/acs.est.1c01359.

(2) Liu, Y.; Han, C.; Ma, J.; Bao, X.; He, H. Influence of Relative Humidity on Heterogeneous Kinetics of NO_2 on Kaolin and Hematite. *Phys. Chem. Chem. Phys.* **2015**, *17* (29), 19424–19431. https://doi.org/10.1039/c5cp02223a.

(3) Wang, C.; Collins, D. B.; Arata, C.; Goldstein, A. H.; Mattila, J. M.; Farmer, D. K.; Ampollini, L.; DeCarlo, P. F.; Novoselac, A.; Vance, M. E.; Nazarof, W. W.; Abbatt, J.P.D. Surface Reservoirs Dominate Dynamic Gas-Surface Partitioning of Many Indoor Air Constituents. *Sci. Adv.* **2020**, *6* (8), 8973. https://doi.org/10.1126/sciadv.aay8973.

(4) Barney, W. S.; Finlayson-Pitts, B. J. Enhancement of N_2O_4 on Porous Glass at Room Temperature: A Key Intermediate in the Heterogeneous Hydrolysis of NO_2 ? *J. Phys. Chem. A* **2000**, *104* (2), 171–175. https://doi.org/10.1021/jp993169b.

(5) Gankanda, A.; Grassian, V. H. Nitrate Photochemistry in NaY Zeolite: Product Formation and Product Stability under Different Environmental Conditions. *J. Phys. Chem. A* **2013**, *117* (10), 2205–2212. https://doi.org/10.1021/jp312247m.

(6) Mack, J.; Bolton, J. R. Photochemistry of Nitrite and Nitrate in Aqueous Solution: A Review. *J. Photochem. Photobiol. A Chem.* **1999**, *128* (1–3), 1–13. https://doi.org/10.1016/S1010-6030(99)00155-0.

(7) Gómez Alvarez, E.; Sörgel, M.; Gligorovski, S.; Bassil, S.; Bartolomei, V.; Coulomb, B.; Zetzsch, C.; Wortham, H. Light-Induced Nitrous Acid (HONO) Production from NO_2 Heterogeneous Reactions on Household Chemicals. *Atmos. Environ.* **2014**, *95* (2), 391–399. https://doi.org/10.1016/j.atmosenv.2014.06.034.
